# Salinity-Induced Physiochemical Alterations to Enhance Lipid Content in Oleaginous Microalgae *Scenedesmus* sp. BHU1 via Two-Stage Cultivation for Biodiesel Feedstock

**DOI:** 10.3390/microorganisms11082064

**Published:** 2023-08-11

**Authors:** Rahul Prasad Singh, Priya Yadav, Ajay Kumar, Abeer Hashem, Graciela Dolores Avila-Quezada, Elsayed Fathi Abd_Allah, Rajan Kumar Gupta

**Affiliations:** 1 Laboratory of Algal Research, Centre of Advanced Study in Botany, Institute of Science, Banaras Hindu University, Varanasi 221005, India; rah.singhbhu@gmail.com (R.P.S.); priya02061995@gmail.com (P.Y.); 2 Amity Institute of Biotechnology, Amity University, Noida 201303, India; 3 Botany and Microbiology Department, College of Science, King Saud University, P.O. Box. 2460, Riyadh 11451, Saudi Arabia; habeer@ksu.edu.sa; 4 Facultad de Ciencias Agrotecnológicas, Universidad Autónoma de Chihuahua, Chihuahua 31350, Mexico; gdavila@uach.mx; 5 Plant Production Department, College of Food and Agricultural Sciences, King Saud University, P.O. Box. 2460, Riyadh 11451, Saudi Arabia; eabdallah@ksu.edu.sa

**Keywords:** *Scenedesmus* sp., biomass, chlorophyll *a* fluorescence, stress biomarkers, flow cytometer, Fourier-transform infrared, nuclear magnetic resonance, triacylglycerides, biodiesel

## Abstract

In the recent past, various microalgae have been considered a renewable energy source for biofuel production, and their amount and extent can be enhanced by applying certain types of stress including salinity. Although microalgae growing under salinity stress result in a higher lipid content, they simultaneously reduce in growth and biomass output. To resolve this issue, the physiochemical changes in microalgae *Scenedesmus* sp. BHU1 have been assessed through two-stage cultivation. In stage-I, the maximum carbohydrate and lipid contents (39.55 and 34.10%) were found at a 0.4 M NaCl concentration, while in stage-II, the maximum carbohydrate and lipid contents (42.16 and 38.10%) were obtained in the 8-day-old culture. However, under increased salinity, *Scenedesmus* sp. BHU1 exhibited a decrease in photosynthetic attributes, including Chl-*a*, Chl-*b*, Fv/Fm, Y(II), Y(NPQ), NPQ, qP, qL, qN, and ETRmax but increased Y(NO) and carotenoids content. Apart from physiological attributes, osmoprotectants, stress biomarkers, and nonenzymatic antioxidants were also studied to elucidate the role of reactive oxygen species (ROS) facilitated lipid synthesis. Furthermore, elemental and mineral ion analysis of microalgal biomass was performed to evaluate the biomass quality for biofuel and cell homeostasis. Based on fluorometry analysis, we found the maximum neutral lipids in the 8-day-old grown culture at stage-II in *Scenedesmus* sp. BHU1. Furthermore, the use of Fourier-transform infrared (FT-IR) and nuclear magnetic resonance (NMR) spectroscopy analyses confirmed the presence of higher levels of hydrocarbons and triacylglycerides (TAGs) composed of saturated fatty acids (SFAs) and monounsaturated fatty acids (MUFAs) in the 8-day-old culture. Therefore, *Scenedesmus* sp. BHU1 can be a promising microalga for potential biodiesel feedstock.

## 1. Introduction

Currently, the increasing global population faces the challenge of accessing energy resources, or fuels. Hence, over the last two decades, the research community has been continuously searching for alternative sources of renewable energy. In this regard, microalgae appear to be a promising alternative for future biofuel feedstock because of their higher photosynthetic activity, rapid generation time, high carbohydrate and lipid content, and capacity to thrive in saline conditions [1,2,3]. In previously published articles, various authors have reported different microalgal species, such as *Chlamydomonas reinhardtii*, *Chlorella vulgaris*, and *Monoraphidium* sp., as having the capability of synthesising the optimum concentration of lipids under laboratory stress conditions [4,5]. However, it is imperative to translate these laboratory findings into industrial-scale applications. In previous studies, it has been well established that the contents of lipids and carbohydrates in microalgal species are significantly enhanced under different stresses, like temperature, pH, light intensity, salinity, and nutrients [6,7]. Although, the implementation of stress must be economically feasible and should have negligible impact on the growth and survival of the algae.

Salt stress is identified as a significant factor that influences the biochemical integrity of microalgae [8,9]. Microalgae can accumulate 20–60% of total lipid per dry cell weight (DCW) depending on strain characteristics and growth conditions [10]. The biofuel production process still has bottlenecks from an economic point of view due to the poor sustainability checks of microalgal biomass and lipids. Exposure to salinity stress induces alterations in the lipid biosynthesis of microalgal cells resulting in the synthesis of neutral lipids, such as TAGs (triacylglycerides). Although major constituents of these TAGs are saturated fatty acids (SFAs) and monounsaturated fatty acids (MUFAs), polyunsaturated fatty acids (PUFAs) are more prevalent in polar lipids [11]. The presence of SFAs and MUFAs in microalgae renders them a promising feedstock for biodiesel because of their high oxidative stability and lower emissions of nitrogen oxides (NO_X_) in comparison to PUFAs. 

In previous studies, various microalgal species, like *Chlorella sorokiniana* and *Desmodesmus asymmetricus* [12], *Chlamydomonas reinhardtii* [13], *Dunaliella salina* [14], and *Scenedesmus quadricauda* [15], have been reported for their enhancement of lipid production under conditions of salinity stress. However, the increase in salinity conditions reduces microalgal growth and biomass productivity. Hence, microalgal cells quickly adapt to stressful conditions by changing their physiology and metabolic processes [16]. For example, *D. salina* adapts to salinity stress by controlling carbon fluxes between glycerol synthesis in the cytoplasm and starch synthesis in the chloroplast [14,17]. However, NaCl-induced salt stress causes the formation of ROS, which triggers cellular macromolecule disintegration [18,19]. Although, salinity stress limits biomass production, which diminishes biofuel yield due to the detrimental impact on growth, chlorophyll content, photosystem II (PSII) proteins, and ribulose-1,5-bisphosphate carboxylase/oxygenase (RuBisCO) enzyme activity [12,17]. To tackle these challenges, researchers have proposed a two-stage cultivation approach as a potential solution. In stage-I, microalgae are grown in nutrient-sufficient conditions. In stage-II, a stress phase is implemented to increase the carbohydrate and lipid contents [20,21,22].

Measuring chlorophyll *a* fluorescence (ChlF) is a non destructive and rapid technique for ascertaining the physiological status of microalgae. It is nonharmful to microalgae cells and offers a plethora of information concerning the functionality of the photosynthetic machinery [23,24]. Many researchers have explored the strategic optimisation of growth parameters to identify strong interactions that reduce biomass losses by drastically increasing the lipid content. Although early research focused on the growth-inhibiting effects of salt stress on biomass, few systematic efforts have been made to understand the impact of salinity stress on the physiochemical parameters of microalgae for biofuel. We adopted two-stage cultivating approach to increase the biomass production and TAGs content.

*Scenedesmus* sp. produces more biomass and lipids under various growing conditions than other green microalgal species [1,25,26]. In the current study, a green microalga, *Scenedesmus* sp. BHU1, was applied in two-stage cultivation under salinity stress for high biomass and lipid accumulation. However, salinity-induced stress is associated with physiological impairment, and its primary target site in the eukaryotic microalgae is not clearly known. Furthermore, in relation to energy-producing molecules, the protective and acclimatisation strategies adapted by microalgae against salinity-induced oxidative damage are still not very clear in view of the inadequate and incomplete knowledge on salinity-induced physiochemical impairment in microalgae at the energy producing level.

In the current study, we evaluated the effect of salinity stress on physiochemical alterations in *Scenedesmus* sp. BHU1 to determine its positive impact on neutral lipid content under two-stage cultivation. Additionally, the effects of NaCl stress on the levels of carotenoids, H_2_O_2_, lipid peroxidation, proline, phenols, and flavonoids were assessed. Further, elemental and mineral analyses (i.e., N, C, H, S, Na^+^, K^+^, Ca^2+^, and Mn^2+^) of microalgal biomass were evaluated to assess the biomass quality for biofuel production and cell homeostasis.

## 2. Materials and Methods

### 2.1. Microalga Species, Culture Medium, and Experimental Design

The microalga *Scenedesmus* sp. BHU1 used in this research was collected from Banaras Hindu University, Varanasi, India, and grown in BG-11N^+^ medium, as previously described [1]. During stage-I, to optimise the concentration of NaCl to determine the maximum growth, carbohydrates, and lipids, microalgal cells were cultivated in BG-11N^+^ medium at 0, 0.05, 0.1, 0.15, 0.2, and 0.4 M NaCl concentrations. All experiments for stage-I cultivation were carried out in the late exponential phase culture (at 14 days). For the stage-II cultivation experiment, microalgal cells were first cultivated in BG-11N^+^ medium for 14 days with a 0 M NaCl concentration (since the highest growth of the test organism was found at this concentration during the stage-I experiment). After, the *Scenedesmus* sp. BHU1 strain was exposed to 0.4 M NaCl for 0, 4, 8, and 12 days during stage-II to induce salt stress (because *Scenedesmus* sp. BHU1 synthesised the maximum carbohydrates and lipids in 0.4 M NaCl during stage-I). 

Each of the experiments were performed in triplicate (*n* = 3) in 1 L conical flasks containing 0.5 L of microalgal culture of 0.1 optical density (OD) from an aseptically growing culture of 0.7 OD according to the N_1_V_1_ = N_2_V_2_ formula, where N_1_ = optical density (OD = 0.7) of the mother culture, V_1_ = desired volume required, N_2_ = desired optical density (OD = 0.1), and V_2_ = volume of experimental culture (0.5 L) in stage-I, and 100% of the actively growing culture of stage-I was inoculated in stage-II. All experiments were carried out in the batch culture at 25 ± 2 °C with a light:dark (12:12 h) photoperiod and 55 μmol photons m^−2^s^−1^ illumination provided by a cool fluorescent tube light at pH 7.4. During the experimental period, to prevent adherence of the microalgae to the wall, the culture flasks were manually shaken 4–5 times daily.

### 2.2. Assessment of Dry Cell Weight and Biomass Productivity

To achieve a constant DCW, 2 mL microalgal culture was centrifuged at 6000 rpm for 5 min in a preweighted Eppendorf tube. The pellet was then washed thrice with deionised water before being oven-dried at 70 °C. The biomass productivity was calculated according to the following equation:

Biomass productivity (mg/L/day) = (X_2_ − X_1_)/(t_2_ − t_1_)

where X_2_ and X_1_ are the DCW (mg/L) at times t_2_ and t_1_, respectively.

### 2.3. Assessment of Photosynthetic Pigment

To determine the amount of pigment present in *Scenedesmus* sp. BHU1, 2 mL microalgal culture was centrifuged at 6000 rpm for 5 min. The methanol was used to dissolve the cell pellet, and then it was kept overnight at 4 °C. Further, the pigment composition (chl-*a*, chl-*b*, and carotenoids) was determined using the following formulas after measuring the absorbance of the supernatant at 665.2, 652.4, and 470 nm, respectively [27].

Chlorophyll *a*: Chl-*a* (µg/mL) = 16.72 × A_665.2_ − 9.16 × A_652.4_

Chlorophyll *b*: Chl-*b* (µg/mL) = 34.09 × A_652.4_ − 15.28 × A_665.2_

Carotenoid (µg/mL) = (1000 × A_470_ − 1.63 × Chl-*a* − 104.9 × Chl-*b*)/221

### 2.4. Analysis of Chlorophyll a Fluorescence (ChlF)

ChlF was used to monitor the photosynthetic activity under a rapid light curve (RLC) using a pulse-amplitude modulation (PAM) KS-2500 apparatus (Heinz Walz GmbH, D-91090, Effeltrich, Germany) connected to a laptop with PamWin 3.20 data recording software. Before any analysis, microalgal cultures were dark-acclimated for 10–15 min. Dark-acclimated cultures (0.5 mL) were pipetted into a steel cuvette with a tiny magnetic stirrer that homogenised the culture during the entire period of measurement.

The dark-adapted minimum ChlF yield (Fo) was determined by delivering low measuring light intensities (<<0.1 µmol photons m^−2^s^−1^) to accelerate any significant difference in fluorescence, and the dark-adapted maximum ChlF yield (Fm) was determined by delivering a 0.3 s saturating pulse at 3000 µmol photons m^−2^s^−1^. A photosynthetic active radiation (PAR) range (5–2973 µmol photons m^−2^s^−1^) was selected for rapid light curve analysis. Then, red actinic light (AL) intensity (104 µmol photons m^−2^s^−1^) was applied to stimulate photochemistry and enable it to reach a steady state (Fs). A second saturating pulse (3000 µmol photons m^−2^s^−1^) was delivered during the induction phase to record the light-adapted maximal ChlF yield (Fm’). Upon completing the induction period and promptly switching off the actinic light, the light-adapted minimal ChlF yield (Fo’) was measured under 5 μmol photons m^−2^s^−1^ of far-red irradiation. The PamWin 3.20 software automatically calculated the maximum photochemical quantum yield (Fv/Fm), effective photochemical quantum yield Y(II), quantum yield of nonregulated energy dissipation Y(NO), quantum yield of regulated energy dissipation Y(NPQ), nonphotochemical fluorescence quenching (NPQ), puddle model-based coefficient of photochemical fluorescence quenching (qP), lake model-based coefficient of photochemical fluorescence quenching (qL), coefficient of nonphotochemical fluorescence quenching (qN), and maximum rate of electron transfer (ETRmax) based on the measured values of Fo, Fm, Fo’, Fm’, and Fs.

### 2.5. Assessment of Protein, Carbohydrate, and Lipid Contents

By making some modifications, the protein content was assessed using the Lowry technique, pioneered in [28]. In brief, 0.5 mL microalgal culture was homogenised in 0.5 mL reagent-A (1N NaOH). After, 2.5 mL freshly prepared reagent-B (10% Na_2_CO_3_, 4% KNaC_4_H_4_O_6_·4H_2_O, and 2% CuSO_4_) was added and incubated at 37 °C for 10 min. The absorbance of the blue colour was measured at 650 nm after the addition of the 1N Folin–Ciocalteu phenol (FCP) reagent. During the determination of the protein content, bovine serum albumin (BSA) was used as a standard.

The anthrone reagent was used to estimate the carbohydrate content using the protocol in [29] with slight modifications. In brief, 0.2 g anthrone was dissolved in 100 mL cold 95% H_2_SO_4_ to prepare the anthrone reagent. Then, 1 mL microalgal culture was pelleted and homogenised in 1 mL 1N NaOH. A total of 100 µL extract was mixed with 4 mL anthrone reagent and maintained a 5 mL volume using 900 µL deionised water. The absorbance of the greenish-blue colour was recorded at 625 nm. During the study, a glucose standard was used for the determination of carbohydrate content.

The Bligh and Dyer technique was used to determine the in vitro lipid content of *Scenedesmus* sp. BHU1 [30]. A total of 0.1 g lyophilised microalgal biomass was homogenised in an extraction solvent of chloroform/methanol/water (2:1:1 *v*/*v*/*v*) and centrifuged at 10,000 rpm for 10 min. The chloroform layer containing the lipid fraction was isolated and washed with a 0.9% NaCl solution (5:1 *v*/*v*). The organic phase was separated and evaporated using a vacuum centrifuge. The following formula was used to calculate the total lipid content gravimetrically:

% Lipid content = lipid weight (g) × 100/lyophilised culture weight (g)

### 2.6. Stress Biomarkers, Osmoprotectant, and Nonenzymatic Antioxidative Assay

A total of 25 mg fresh cells of *Scenedesmus* sp. BHU1 was homogenised in a 0.1% trichloroacetic acid solution to measure the amount of H_2_O_2_. From the centrifuged homogenate, 0.5 mL supernatant was agitated with 0.5 mL 0.01 M phosphate buffer (pH 7.0) and 1 mL 1 M KI. The absorbance of the solution was measured at a 390 nm wavelength. The H_2_O_2_ standard was used to estimate the H_2_O_2_ concentration. The H_2_O_2_ content was expressed as µmol mg^−1^ fresh cell weight [31].

Lipid peroxidation was quantified using thiobarbituric acid (TBA) and demonstrated to be equivalent to malondialdehyde [32]. A total of 25 mg fresh microalgal cells was homogenised in a 5% trichloroacetic acid (TCA) solution. From the centrifuged homogenate, 1 mL supernatant was agitated with 1 mL 0.65% TBA made in a 20% TCA solutionå and then warmed at 95 °C for 25 min. After cooling at ambient temperature, the solution was centrifuged at 10,000 rpm for 10 min. The absorbance of the supernatant was measured at 600, 532, and 450 nm wavelengths. The following equation was used to determine the malondialdehyde (MDA) content [33]:

MDA (µmol mg^−1^ fresh cell weight) = [6.45 × (A_532nm_ − A_600nm_)] − [0.56 × A_450nm_]/fresh cell weight (mg)

The procedure outlined in [34] was utilised to measure the proline content in *Scenedesmus* sp. BHU1. A total of 25 mg fresh microalgal cells was homogenised in 3% (*w*/*v*) sulfosalicylic acid. The resultant homogenate was centrifuged, and 1 mL supernatant was mixed with 1 mL freshly prepared acidic ninhydrin, 1 mL acetic acid glacial, and heated at 100 °C for 1 h. The reaction was stopped by placing the sample in an ice bath. After extracting the pink-coloured product in 2 mL toluene, the absorbance was measured at 520 nm. A proline standard was used to estimate the proline concentration. The proline level was expressed as µmol mg^−1^ fresh cell weight.

The total phenolic content (TPC) of 25 mg of fresh cells of *Scenedesmus* sp. BHU1 was extracted in 70% acetone by applying the Folin–Ciocalteu phenol (FCP) reagent technique [35]. The extracting solution was centrifuged at 6000 rpm for 10 min. Then, 1 mL supernatant, 1 mL 1N FCP, and 2 mL 2% Na_2_CO_3_ were mixed and maintained at a 10 mL final volume using deionised water. The solution mixture was heated for 20 min to develop a blue colour due to the reduction of the Folin reagent. The mixture was then allowed to cool at ambient temperature. The absorbance of the blue colour was taken at 760 nm, and the total phenolic content was expressed in µg gallic acid equivalent (GAE)/mg of the fresh cell weight. A gallic acid standard was used for the estimation of the TPC.

The total flavonoid content (TFC) of the 25 mg of fresh cells of *Scenedesmus* sp. BHU1 was measured using the aluminium chloride (AlCl_3_) colorimetric technique [36]. A total of 1 mL supernatant was agitated with 1 mL 2% AlCl_3_ and vortexed thoroughly. The solution was kept at ambient temperature for 60 min. The absorbance of the golden yellow colour was taken at 420 nm. The quercetin standard was used for the estimation of TFC. TFC was expressed as µg quercetin (QE)/mg of fresh cell weight.

### 2.7. Elemental and Mineral Composition Analysis of Biomass

The CHNS content in the lyophilised biomass of *Scenedesmus* sp. BHU1 was determined using a EuroVector Elemental Analyzer (EuroEA 3000, Pavia, Italy). Helium and oxygen (99.995% purity) were used as the carrier combustion gases. The combustion capsule was operated at a temperature of 980 °C and an oven temperature of 105 °C. The loaded samples (1 mg) were weighed in a combustion capsule using an auto-balance Mettler-Toledo (WXTS3DU, Zurich, Switzerland). Sulfanilamide (C-41.855%, H-4.676%, N-16.267%, and S-18.618%) was used as a standard.

The mineral content (Na^+^, K^+^, Ca^2+^, and Mn^2+^) was quantified by employing the tri-acid technique, as described in [37] with slight modifications. A total of 50 mg lyophilised microalgal biomass was mixed with conc. HNO_3_:H_2_SO_4_:HClO_4_ (5:1:1 *v*/*v*/*v*) and left overnight. The sample mixture was heated on a hot plate for digestion until it was colourless. The digest was allowed to cool at ambient temperature, and then the contents were diluted to an appropriate volume and filtered through Whatman filter paper. Inductively coupled plasma-mass spectrometry (ICP-MS) was used to analyse mineral ions (Perkin Elmer, Optima 7000 DV, Waltham, MA, USA).

### 2.8. In Vivo Neutral Lipid Analysis through a Flow Cytometer and Fluorescent Microscopy

Nile Red (2 µL/mL) from stock solution (0.25 mg/mL acetone) was mixed with microalgal samples and incubated in the dark for 10 min. Flow cytometry analyses were carried out using a CytoFLEX LX Beckman Coulter (BC47041, Pasadena, CA, USA) flow cytometer equipped with a 561 nm, 30 mW solid-state diode laser. The sample cells were individually captured by the laser beam (flow rate: 30 µL/min) after being entrained in the core of the sheath fluid, resulting in scattering and fluorescence signals. The voltage was calibrated for various light wavelengths in order to examine the cell subpopulations. For each flow cytometry analysis, the forward scatter (FSC) and sideward scatter (SSC) signals were used to assess the cell size, structure, and granularity. The following fluorescence channels were applied for chlorophyll and Nile red fluorescence, respectively: a 585/42 band-pass filter with CytoFLEX channel Y585-PE-A excited at 633 nm and a 763/43 band-pass filter with CytoFLEX channel Y763-PC-7-A excited at 561 nm. The data from the flow cytometry were analysed using the offline software CytExpert 2.3 (Beckman Coulter, Pasadena, CA, USA). The data obtained from the flow cytometer (FCM) are expressed in percentage (%).

For fluorescence microscopy analysis, 2 mL microalgal culture was centrifuged and washed thrice with 1X phosphate buffer saline (PBS). 5 µL microalgal cell suspensions were mixed with 2 µL Nile Red on the slide centre and incubated in darkness for 5 min. The stained cells were examined using a fluorescent microscope with a 20X objective lens (Nikon ECLIPSE 90i, Aurora, CO, USA). The microscope imaging software NIS-Elements AR 4.0 (Nikon Instech Co., Ltd., Tokyo, Japan) was used for the red channel excitation-emission wavelengths at 590 and 650 nm.

### 2.9. Functional Group Characterisation of Biomass by FT-IR Spectroscopy

A total of 0.5 mg lyophilised biomass of *Scenedesmus* sp. BHU1 was mixed with 5 mg KBr and compressed into a disc. Infrared absorption spectra were recorded using a Spectrum Two FT-IR Spectrometer (PerkinElmer, Waltham, MA, USA), with an average of 16 scans and a spectral resolution of 4 cm^−1^, spanning a wavenumber range of 400–4000 cm^−1^. To correct the baseline, all spectra were processed using IR solution software version 10.4.3. The functional groups of proteins, carbohydrates, and lipids (including hydrocarbons and TAGs) were quantified by analysing the peak intensity at different wavenumbers (cm^−1^) of each biomolecule in the spectra.

### 2.10. Oil and Biodiesel Analysis using NMR Spectroscopy

The conversion of fatty acids into methyl esters of fatty acids (FAMEs) through transesterification was carried out using a modified protocol of that found in [38]. To create FAMEs, the extracted lipids were combined with a 2 mL solution of 3% H_2_SO_4_ in methanol. The reaction was conducted in a water bath at a constant temperature of 70 ± 1 °C for 4 h. After the esterification process, 2 mL n-hexane (HPLC grade) was introduced into the reaction mixture and allowed to stand for 4 h. Subsequently, the upper layer, which consisted of n-hexane, was separated and used for NMR analysis to identify the fatty acid methyl ester (FAME) functional groups. The ^1^H NMR spectroscopy analysis of *Scenedesmus* sp. BHU1 oil and biodiesel samples was performed using CDCl_3_ as the solvent.

### 2.11. Statistical Analysis

Averages of three biological replicates (*n* = 3) with a standard error of mean (SEM) were used to present the outcomes. Scientific data analysis software SPSS 21.0 (IBM Corp., New York, NY, USA) was used to apply one-way analysis of variance (ANOVA) to identify the significant effect of salinity stress and its interactive effect on *Scenedesmus* sp. BHU1 parameters. Tukey’s post hoc test with a probability threshold of *p* < 0.05 was used to determine the level of significant differences among treatments. Moreover, to gain a better understanding of the correlation among different physiological, biochemical, stress biomarkers, and elemental parameters under two-stage cultivation, we employed the Pearson correlation to draw a correlation plot using the graphing and analysis software Origin Pro 2023 (Origin Lab Corporation, Northampton, MA, USA). The graphs were drawn using the scientific software GraphPad Prism version 8.0.2 (San Diego, CA, USA) and Origin Pro 2023.

## 3. Results and Discussion

### 3.1. Salinity-Induced Effect on Dry Cell Weight (DCW) and Biomass Productivity (BP) of Scenedesmus *sp.* BHU1

**Figure 1:**
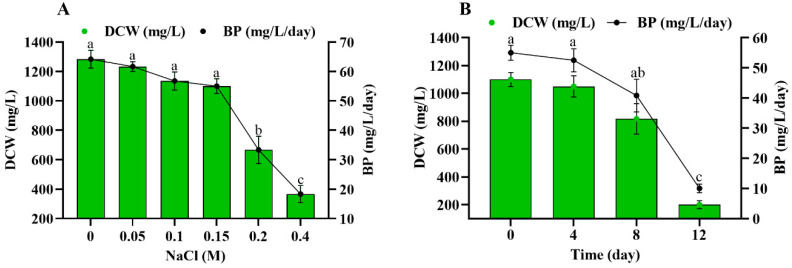
Effect of different concentrations of NaCl (0–0.4 M) during stage-I (**A**) and salinity-induced stress (0.4 M NaCl) for different durations during stage-II (**B**) on dry cell weight (DCW) and biomass productivity (BP) of *Scenedesmus* sp. BHU1. Bars are the mean ± standard error of the mean (SEM) of three biological replicates (*n* = 3). Different lowercase alphabet letters (a–c) are used above the bars for both DCW and BP to indicate the level of statistically significant difference, and the values followed by the same alphabet letters refer to the statistically nonsignificant difference among treatment means at the *p* < 0.05 probability threshold (Tukey’s post hoc test).

The impact of salinity stress was studied in two-stage cultivation to assess the growth in terms of DCW and biomass productivity. The NaCl addition in two-stage salinity stress significantly (*p* < 0.05) influenced the DCW and biomass productivity of *Scenedesmus* sp. BHU1. During stage-I, 0–0.4 M NaCl supplementation reduced the DCW and biomass productivity of *Scenedesmus* sp. BHU1 (Figure 1A). The current study found a positive relation between DCW and biomass productivity during stage-I. The 0 M NaCl culture had the highest DCW and biomass productivity, which were both 3.4-fold higher than 0.4 M NaCl-grown cultures. Similar research found a downward trend in the DCW and biomass productivity of *S. quadricauda*, *Scenedesmus dimorphus*, *Chlorella* sp., and *Acutodesmus dimorphus* when exposed to NaCl concentrations ranging from 0.08 to 0.32 M and 0.02 to 0.2 M, respectively [2,39]. Significant decreases in DCW and biomass productivity were recorded in the 0.2 and 0.4 M NaCl treated cultures in stage-I due to the increase in the number of intracellular Na^+^ ions [19,21]. However, there was an insignificant effect of NaCl from 0–0.15 M on the DCW and biomass productivity (Figure 1A). According to previous research, the highest DCW and biomass productivity of *Scenedesmus* sp. CCNM 1077 and *Tetraselmis subcordiformis* were found in 0 and 0.09 M NaCl-grown cultures, respectively [21,40]. When *Anabaena fertilissima* cells exposed to 0.25–0.5 M NaCl, the intracellular Na^+^ content increased, leading to an alkaline solution that triggered a rapid rise in pH, which is lethal for microalgal growth [41]. Furthermore, it has been observed that low concentrations of NaCl are advantageous for certain metabolic processes that promote the growth of microalgae, but excessive concentrations result in the death of microalgae [13,42,43,44]. The dynamic equilibrium between ROS production and consumption is disturbed by excess salt ions, leading to cell death [45]. The altered osmotic potential of cells and cell membrane permeability because of membrane-specific ion channels, water potential, ion transport, CO_2_, and O_2_ solubility were additional causes of changes in cellular ionic ratios that resulted in growth inhibition [46].

*Scenedesmus* sp. BHU1 was first cultivated in BG-11N^+^ culture medium for 14 days before being subjected to stage-II salinity stress, which was applied by adding 0.4 M NaCl for 0, 4, 8, and 12 days of cultivation. Stage-II salinity stress was given at different time intervals (days) that affected the DCW and biomass productivity of *Scenedesmus* sp. BHU1. The findings demonstrate that during stage-II cultivation, exposure to salt stress for 0, 4, 8, and 12 days resulted in a gradual decrease in DCW and biomass productivity (Figure 1B). The findings revealed a significant positive correlation between DCW and biomass productivity during stage-I and -II salinity-induced stress (Figure S1A,B). Similarly, *Scenedesmus* sp. CCNM 1077 and *Desmodesmus abundans* have exhibited a decrease in biomass in two-stage salt stress cultivation [21,47]. Interestingly, stage-II salinity stress did not significantly decrease cellular biomass production from 0 to 8 days, suggesting an effective strategy to produce more carbohydrates and lipids for biofuel feedstock without reducing biomass production.

### 3.2. Salinity-Induced Effect on Morphological Changes of Scenedesmus *sp.* BHU1

**Figure 2:**
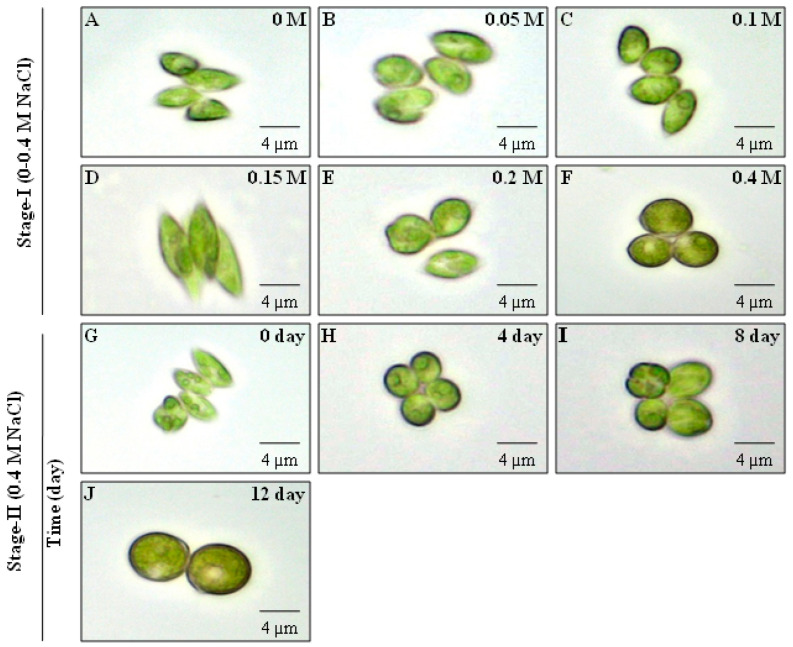
Effect of different concentrations of NaCl (0–0.4 M) during stage-I (**A**–**F**) and salinity-induced stress (0.4 M NaCl) for different durations during stage-II (**G**–**J**) on the morphological changes of *Scenedesmus* sp. BHU1. The image was captured at a 40X magnification using a light microscope (CX21iLEDFS1, Olympus).

*Scenedesmus* sp. is frequently found in both freshwater and wastewater systems. It primarily manifests as cells in multiples of two, four, and eight, exhibiting distinct morphological phenotypes. The main differences between *Scenedesmus* spp. are the cell number and spines formed in coenobium, as well as the texture of the cell wall. *Scenedesmus* sp. is a pleomorphic chlorophycean green microalga that undergoes morphological modifications that separate it from coenobium in response to increased salt stress. In the current study, it was observed that salt stress causes morphological alterations in *Scenedesmus* sp. BHU1 during stage-I and -II cultivation (Figure 2). In stage-I, the maximum cell size was observed in 0.4 M NaCl-supplemented culture, which was larger than the 0 M NaCl grown culture (Figure 2A,F). This result unequivocally demonstrated that *Scenedesmus* sp. BHU1 cells become larger and more spherical under salinity stress. A similar pattern was also found in *D. salina* under salt stress [14]. Additionally, *Scenedesmus* sp. BHU1 cells cultured in 0–0.15 M NaCl continue to form coenobia of four-spindle-shaped cells organised alternately to a slightly linear manner (Figure 2A–D). The high salt content (0.2 and 0.4 M NaCl) caused the destruction of the apex of the spines in all cells of coenobium. Consequently, the cells became ellipsoidal to ovoid during stage-I (Figure 2E,F). The cells became spherical and larger during stress, because they synthesised more carbohydrate, lipid, H_2_O_2_, malonaldehyde, and proline [14,48]. The ability of *Scenedesmus* sp. BHU1 to synthesise a high carbohydrate and lipid content makes it one of the most prominent microalga for biofuel feedstock. *Scenedesmus* sp. BHU1, during stage-II cultivation with 0.4 M NaCl at different durations, followed a similar pattern to stage-I cultivation (Figure 2G–J). The substantial increase in size due to the lipid and other macromolecule synthesis in *Scenedesmus* sp. BHU1 may explain the notable phenotypic alterations.

### 3.3. Salinity-Induced Effect on Pigment Contents of Scenedesmus *sp.* BHU1

The thylakoid membrane serves as the structural framework for the chloroplast, making it essential for the absorption and conversion of photon energy into chemical energy [49,50]. A crucial marker of the photosynthetic potential of plants and microalgae is the existence of photosynthetic pigments [45]. To evaluate the impact of salt stress on the growth of *Scenedesmus* sp. BHU1, the pigment content was monitored during two-stage cultivation. Osmotic and ionic stress caused by the increase in NaCl supplementation in the culture resulted in a significant (*p* < 0.05) reduction in chl-*a* and chl-*b* contents [8]. In our investigation, the maximum levels of chl-*a* and chl-*b* were found in 0 M NaCl, while the minimum levels of chl-*a* and chl-*b* were found in 0.4 M NaCl in stage-I (Table 1). Similar to our findings, microalgae, such as *C. reinhardtii*, Scenedesmus sp. CCNM 1077, and *Scenedesmus obliquus* XJ002, have shown decreased photosynthetic pigment under salt stress [19,21,45]. Furthermore, supplementing *C. reinhardtii* with 0 to 0.2 M NaCl reduced chl *a* + *b* levels from 14 to 9 g/mL [51]. Reduced photosynthetic pigment content could be a sign of oxidative stress caused by increased chlorophyllase activity and reduced RuBisCo activity because of poor CO_2_ uptake that encourages pigment deterioration [13,45]. It has been noted that salinity stress has a deleterious impact on carbon fixation and carbon concentrating processing *C. reinhardtii*, which are necessary for the availability of CO_2_ for RuBisCo activity [13]. Another cause of lowering photosynthetic efficiency is that a higher quantity of ROS is synthesised in cells, which lower photosynthetic pigment through the peroxidation of thylakoid membrane lipid and degradation of PSII complex [52]. The decrease in chl-*a* and chl-*b* levels would reduce photosynthetic capacity and ultimately inhibit the growth of microalgal cells. However, carotenoids and the carotenoids/total chl ratio significantly (*p* < 0.05) increased with the elevated salt stress; this is an indirect marker of salinity stress. In contrast, green microalgae accumulate carotenoids as antioxidant molecules to protect against ROS damage and lipid peroxidation under unfavourable conditions [42,45]. In the current research, carotenoids and the carotenoids/total chl ratio in 0.4 M NaCl were 23- and 118-fold higher, respectively, than 0 M NaCl grown cultures during stage-I. According to [19], carotenoids are essential for cell growth because they scavenge ROS, reduce membrane lipid peroxidation and protect the photosynthetic apparatus against stress responses.

Stage-II salinity stress had a similar effect as stage-I salinity stress in that it decreased the photosynthetic activity of *Scenedesmus* sp. BHU1, resulting in a significant decrease in the quantity of photosynthetic pigment (Table 1). Approximately 4.9- and 2.4-fold decreases in chl-*a* and chl-*b* were measured at 12 days compared to 0 day cultures, respectively. Similar trends in chl-*a* and chl-*b* during stage-II cultivation suggest that *Scenedesmus* sp. BHU1 maintains nearly identical photosynthetic efficiency as stage-I. Similar to our findings, [21] also reported an identical photosynthetic pigment pattern in *Scenedesmus* sp. CCNM 1077 under stage-II salinity stress. In the current study, significant (*p* < 0.05) differences in the carotenoids and carotenoids/total chlorophyll contents were observed, which were 5.9- and 21.6-fold higher at 12 days compared to day 0 in stage-II cultivation. This finding suggests that carotenoids play a protective role during salinity-induced oxidative damage. According to the results of the current investigation, there was a negative correlation between chl-*a* and carotenoids, while a positive correlation existed between chl-*a* and chl-*b* under stage-I and -II salinity stress (Figure S1A,B). Based on the above findings, overall, our results show that salt stress decreased chl-*a* and chl-*b* contents in *Scenedesmus* sp. BHU1, thereby reducing the activity of PSII photochemistry.

### 3.4. Salinity-Induced Effect on Cell Health in Terms of Photosynthetic Performance of Scenedesmus *sp.* BHU1

**Figure 3:**
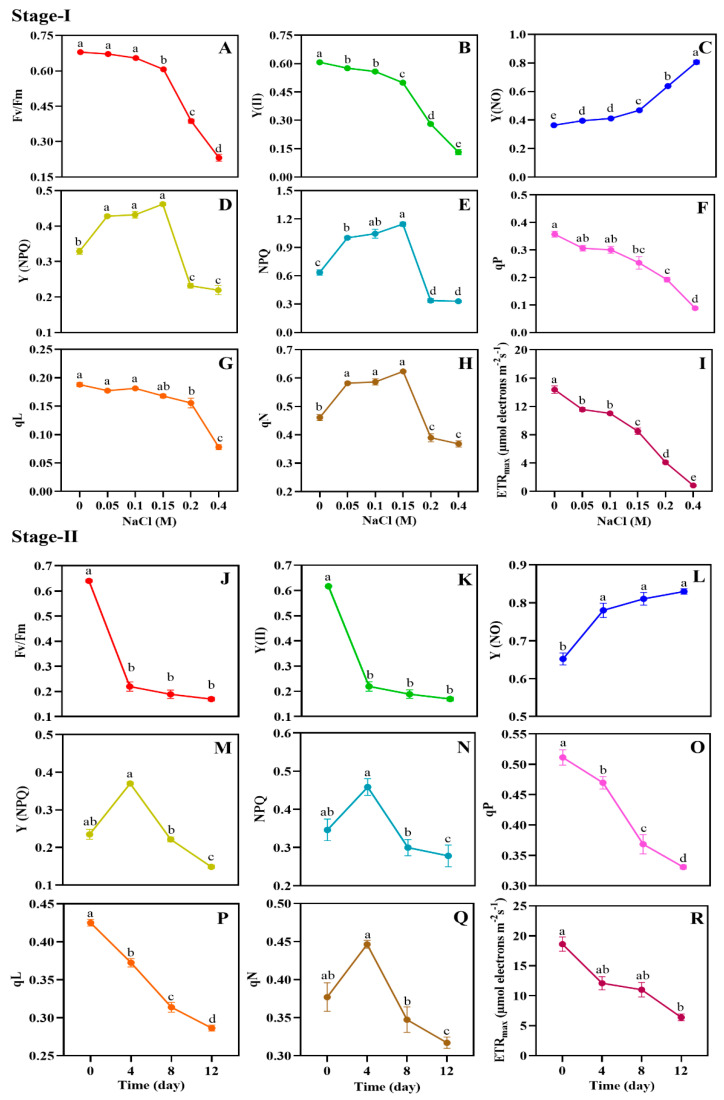
Effect of different concentrations of NaCl (0–0.4 M) during stage-I (**A**–**I**) and salinity-induced stress (0.4 M NaCl) for different durations during stage-II (**J**–**R**) on photosynthetic activity resulting from chlorophyll *a* fluorescence measurement by rapid light curves (RLC) in *Scenedesmus* sp. BHU1. (**A**,**J**) PSII maximum photochemical quantum yield (Fv/Fm); (**B**,**K**) PSII effective photochemical quantum yield Y(II); (**C**,**L**) PSII nonregulated energy dissipation quantum yield Y(NO); (**D**,**M**) PSII regulated energy dissipation quantum yield Y(NPQ), (**E**,**N**) nonphotochemical fluorescence quenching (NPQ); (**F**,**O**) coefficient of photochemical fluorescence quenching based on the puddle model (qP); (**G**,**P**) coefficient of photochemical fluorescence quenching based on the lake model (qL); (**H**,**Q**) nonphotochemical fluorescence quenching coefficient (qN); (**I**,**R**) maximum electron transfer rate (ETRmax). Bars are the mean ± standard error of the mean (SEM) of three biological replicates (*n* = 3). Different lower-case alphabet letters (a–e) above the bars indicate the level of statistically significant difference, and the values followed by the same alphabet letters refer to the statistically nonsignificant difference between treatment means at the *p* < 0.05 probability threshold (Tukey’s post hoc test).

Pulse-amplitude modulated (PAM) fluorometry is a commonly employed, rapid, and noninvasive technique for evaluating photosynthetic activity in microalgae. ChlF measurement using PAM is an effective photosynthetic biomarker for monitoring and assessing the effect of salinity stress on the growth of microalgae. The physiological activity of *Scenedesmus* sp. BHU1 under salinity stress over two-stages (stage-I and -II) was thoroughly documented throughout the entire research period (Figure 3). In stage-I, microalgal cells subjected to NaCl concentrations, ranging from 0 to 0.4 M, exhibited a significant (*p* < 0.05) reduction in Fv/Fm, Y(II), qP, qL, and ETRmax. This suggests that the photosynthetic activity of *Scenedesmus* sp. BHU1 is significantly affected by the presence of high salt content. The highest Fv/Fm, Y(II), qP, qL, and ETRmax were recorded in 0 M NaCl-grown cultures compared to 0.4 M NaCl-grown cultures (Figure 3A,B,F,G,I). Likewise, similar trends in the salinity-induced reduction of photosynthetic activity in *C. vulgaris* and *C. sorokiniana* are already documented [16,53]. The notable decrease in Fv/Fm and Y(II) indicates that the NaCl stress led to a decline in the capacity of PSII and an inefficient conversion of absorbed photon energy into electron energy. This occurred as a result of poor PSII repair and decreased turnover of the D1 protein [16,54]. Additionally, the decrease in qP and qL (Figure 3F,G), which represent the fraction of an “open” reaction centre, demonstrated that the reaction centre was relatively closed after NaCl treatment, which further decreased the conversion of photochemistry and linear electron flow [55]. The light curves revealed that salt stress drastically reduced the values of ETRmax (Figure 3I), indicating a reduction in the light response of PSII. The decreased ETRmax is the result of the transition from linear electron flow (LEF) to cyclic electron flow (CEF), which may serve to protect PSII from excessive activation energy [49].

Moreover, during stage-I, there was a gradual rise in Y(NO) from 0 to 0.4 M NaCl (Figure 3C), indicating an accelerated increase in the proportion of heat dissipation from the PSII reaction centre and the PSII’s capacity to protect itself from salt-induced damage [56]. This implies that the nonphotochemical process of energy dissipation was effective, and excess excitation energy was able to be effectively transformed into heat. Conversely, Y(NPQ), NPQ, and qN (Figure 3D,E,H) exhibited increases up to 0.15 M NaCl during stage-I. However, they subsequently displayed a decreasing trend from 0.2 to 0.4 M NaCl. This demonstrates that nonregulated (Y(NO)) quenching processes in PSII lost more energy flux absorption to defend against overreduction of the electron transport chain (ETC) in stressed *Scenedesmus* sp. BHU1. These results suggest that the impact of regulated heat dissipation in *Scenedesmus* sp. BHU1 on protecting the photosynthetic machinery may be comparatively less significant than that of nonregulated heat dissipation. Comparable outcomes were also documented for *D. salina* and *C. vulgaris* when subjected to salt stress [14,16].

Salinity-induced stress during stage-II resulted in a decrease in the photosynthetic efficiency of *Scenedesmus* sp. BHU1. When comparing a 12-day grown culture to a culture grown for 0 days, reductions were observed in Fv/Fm, Y(II), qP, qL, and ETRmax (Figure 3J,K,O,P,R). A similar pattern of Fv/Fm, Y(II), qP, qL, and ETRmax during stage-II salinity stress demonstrates that *Scenedesmus* sp. BHU1 retained nearly similar photosynthetic activity as in stage-I. Furthermore, increased Y(NO) (Figure 3L) and decreased Y(NPQ), NPQ, and qN (Figure 3M,N,Q) indicate that greater amounts of absorbed energy flux were dissipated in PSII via nonregulated quenching mechanisms. The salt course reduction of NPQ reflects the thylakoid lumen acidification [57]. The decreased NPQ in stressed cultures suggests that it could not induce sufficient xantophyll cycle activation and ΔpH-dependent heat dissipation [57]. In both stage-I and stage-II cultivations of the present study, Fv/Fm, Y(II), qP, qL, and ETRmax exhibited positive correlations with each other, while Y(NO) showed negative correlations with these parameters (Figure S1A,B). These findings suggest that the PSII of *Scenedesmus* sp. BHU1 is highly sensitive to salt-induced oxidative damage. The aforementioned findings led to the conclusion that the microalgae *Scenedesmus* sp. BHU1 undergoes diverse physiological changes by altering the production of various cell components.

### 3.5. Salinity-Induced Effect on Biochemical Contents of Scenedesmus *sp.* BHU1

**Figure 4:**
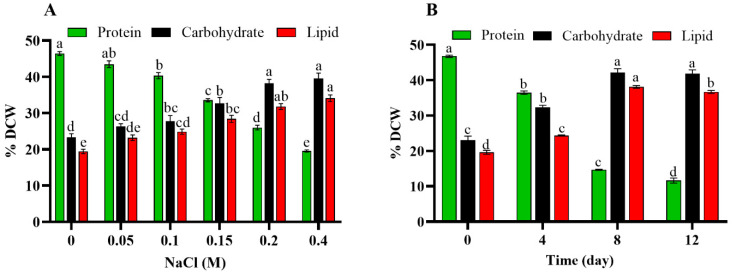
Effect of different concentrations of NaCl (0–0.4 M) during stage-I (**A**) and salinity-induced stress (0.4 M NaCl) for different durations during stage-II (**B**) on the biochemical content of *Scenedesmus* sp. BHU1. Bars are the mean ± standard error of the mean (SEM) of three biological replicates (*n* = 3). Different lowercase alphabet letters (a–e) above the bars indicate the level of statistically significant differences, and the values followed by the same alphabet letters refer to the statistically nonsignificant differences among treatment means at the *p* < 0.05 probability threshold (Tukey’s post hoc test).

The biochemical content (protein, carbohydrate, and lipid) of *Scenedesmus* sp. BHU1 was found to be altered by varying NaCl concentrations during stage-I and varying time intervals during stage-II (Figure 4). The cellular protein in *Scenedesmus* sp. BHU1 was significantly (*p* < 0.05) reduced by salinity stress. This reduction was similar to that shown in biomass productivity, photosynthetic pigment, and photosynthetic efficiency. The culture grown with 0 M NaCl had the highest protein content (46.44 ± 0.54%), which was 2.3-fold higher than the protein content (19.54 ± 0.31%) of 0.4 M NaCl-grown culture (Figure 4A). Likewise, similar outcomes were also noted in other green microalgae, such as *C. reinhardtii* and *A. dimorphus* [19,39]. The protein content of the 0 day culture was 46.75 ± 0.54% in stage-II cultivation, whereas 4-, 8-, and 12-day-stressed cultures showed a significant decline in protein content (Figure 4B). The reduction in protein levels during salt stress may be primarily attributed to the downregulation of key genes involved in protein synthesis, autophagy, and degradation processes [21,39]. 

In microalgal cells under stress, the photosynthetic carbon flow directs the metabolic energy towards the synthesis of diverse energy-rich molecules. Under salinity stress, carbohydrates and lipids showed a positive correlation, while a negative correlation was found between carbohydrate and protein (Figure S1A,B). The quantity of carbohydrate and lipid increased significantly (*p* < 0.05) from 23.31 ± 1.01% and 19.43 ± 0.60% to 39.55 ± 0.53% and 34.10 ± 0.90% under 0 and 0.4 M NaCl supplementation, respectively (Figure 4A). Similar results were also noted in other green microalgae, such as A. dimorphus [39], *Tetraselmis suecica* [58], *S. quadricauda*, *S. dimorphus*, *Chlorella* sp. [2], and *C. reinhardtii* [19]. According to some studies, microalgae under extreme salt stress accumulate more carbohydrates than lipids [51]. In our study, the lipid content (34.10 ± 9.07%) of *Scenedesmus* sp. BHU1 was 1.1-fold lower compared to carbohydrate content (39.55 ± 0.53%) in 0.4 M NaCl concentration. Under salt stress, microalgae utilise carbohydrates as osmoprotectants to ensure osmotic adjustment and homeostasis [59,60], and it also increases cell adaptation ability [8]. Previous research has suggested that the elevated lipid content may be due to the simultaneous conversion of surplus carbohydrates into lipids by metabolic pathways [13,51]. This increase in lipids maintains the integrity of the membrane in response to salt stress, which reduces the osmotic pressure of the cell membrane [45].

Similar to stage-I salinity stress, stage-II also considerably increased the carbohydrate and lipid contents in *Scenedesmus* sp. BHU1. The 8-day salt-stressed cultures had the highest carbohydrate and lipid contents (42.16 ± 1.08 and 38.10 ± 0.37%) compared to 12-day (41.80 ± 1.11 and 36.66 ± 0.43%) cultures (Figure 4B). During stage-II, 12-day salt-stressed microalgal cells were associated with lower carbohydrate and lipid synthesis than 8-day salt-stressed cells. One of the potential causes of this could be that under high salinity for a short period, cell respiration activity increases, leading to disintegration of energy-rich storage molecules like carbohydrates and lipids [21,39]. Overall, our finding implies that under high salt stress, the internal lipid of microalgae was enhanced and may serve as a promising energy material for future energy storage in the bioenergy industry. 

### 3.6. Salinity-Induced Effect on Stress Biomarkers, Osmoprotectant, and Nonenzymatic Antioxidant Content of Scenedesmus *sp.* BHU1

Under adverse conditions, microalgae produce a variety of ROS, including Superoxide ion (O_2_^•–^), hydroxyl radicals (HO^•^), and hydrogen peroxide (H_2_O_2_). These ROS are extremely lethal and damage lipids, proteins, DNA, and other cellular macromolecules, inhibiting cell growth and ultimately leading to cell death. Plants and microalgae possess various inherent defence mechanisms to protect cells from ROS damage, including osmoprotectant (proline), nonenzymatic molecules (TPC and TFC), and enzymatic molecules like CAT and APX [19,21,33].

In the present study, the effect of salt stress on ROS production was assessed by monitoring the level of H_2_O_2_. The results reveal that an increase in NaCl caused a statistically significant (*p* < 0.05) increase in H_2_O_2_ accumulation in *Scenedesmus* sp. BHU1. The highest concentration of H_2_O_2_ was recorded in 0.4 M NaCl-cultivated cells (Table 2). This H_2_O_2_ concentration was 4.0-fold higher than that of the cells grown in 0 M NaCl culture. Similar observations were also made in two green microalgae, *C. reinhardtii* and *C. vulgaris*, during salinity stress [19,61]. ROS generation may cause changes in microalgal metabolism, including decreased nutrient intake, reduced CO_2_ flux, photoreduction, thylakoid membrane lipid peroxidation, and the production of triplet chlorophyll [19]. On the other hand, H_2_O_2_ synthesis is required for the phytochemicals abscisic acid (ABA) and brassinosteroids (BRs), which improve stress tolerance in plants and microalgae [43]. Increased stress duration during stage-II cultivation significantly increased H_2_O_2_ levels in *Scenedesmus* sp. BHU1. The cultures under stress for 12 days and 8 days had the highest levels of H_2_O_2_, which were 5.8- and 5.1-fold greater compared to 0 day culture, respectively. An insignificant increase in the H_2_O_2_ concentration was observed in the 4-day stressed culture as compared to the 0 day culture. The unchanged metabolic activities of *Scenedesmus* sp. BHU1 are the main cause of the insignificant (*p* > 0.05) increase in H_2_O_2_ level in 4 days of stressed culture.

Another stress biomarker frequently employed in microalgae is lipid peroxidation, which is assessed by the cell’s MDA concentration [39,59]. MDA is a major consequence of lipid peroxidation, which is caused by free radicals oxidising polyunsaturated fatty acids. Membrane lipid peroxidation is a primary indicator of cell damage; therefore, it is considered an oxidative stress biomarker [62,63]. Similar to the H_2_O_2_ results, the MDA contents also significantly (*p* < 0.05) increased in 0.4 M NaCl, which was 2.5-fold higher than the 0 M NaCl-grown culture (Table 2). However, its level increased nonsignificantly from 0 to 0.2 M in the NaCl-supplemented cultures. Similar to the current research, biofuel-producing chlorophycean microalgae, including *A. dimorphus* and *Chlorella vulgaris*, accumulated the highest amounts of MDA content [39,46]. The microalgae *Scenedesmus* sp. BHU1 showed signs of oxidative stress due to elevated levels of MDA caused by membrane lipid peroxidation during salinity stress. Numerous investigations have documented MDA synthesis in microalgal cells as a result of lipid peroxidation in salt-stressed conditions [19,43,64]. Stage-II showed a negligible increase in MDA levels on 4-day-stressed cultures compared to 0-day-grown cultures. However, 8- and 12-day-stressed cultures had significantly higher MDA levels compared to the 0-day-grown culture (Table 2). The notable increase in MDA concentration at 8 and 12 days during stage-II can be attributed to the accumulation of neutral lipids in *Scenedesmus* sp. BHU1. This explanation suggests that MDA, which is the oxidative by-product of lipid peroxidation, was elevated due to the higher levels of neutral lipids [14,21].

Osmoprotectants, nonenzymatic, and enzymatic antioxidant molecules form the defence mechanism of microalgae, which are highly activated in response to stress [59,65]. In the current study, total phenolics, total flavonoids, and proline were examined as nonenzymatic and osmoprotectant scavengers. Proline is a crucial amino acid that serves various purposes in response to stress, such as maintaining cytosolic pH, functioning as a compatible solute, scavenging ROS, and acting as a chaperone molecule to maintain the stability of protein structures [19]. It is the molecule that is most often accumulated as a stress indicator to prevent cell harm from ROS [60,66]. In the current investigation, *Scenedesmus* sp. BHU1 had significantly (*p* < 0.05) more proline accumulation under 0.2 M NaCl, which was 1.9-fold higher than 0 M NaCl grown culture (Table 2). The biofuel producing microalgae, including *Parachlorella kessleri*, *D. salina*, and *A. dimorphus*, had higher proline levels under salt stress conditions [3,14,39]. During stage-II salinity stress, the highest proline content was found on day 8, while the minimum was found on day 0 (Table 2). Therefore, a dose-dependent increase in proline concentration indicates its adaptive response to salinity stress.

The TPC and TFC are potent ROS scavengers that improve the integrity of the cell membrane and insulate cells against oxidative damage [19,67]. They function as antioxidant molecules, like proline, assisting in osmotic equilibrium and free radical scavenging to lessen the adverse consequences of salt stress [65,68]. In the current investigation, *Scenedesmus* sp. BHU1 showed a significant (*p* < 0.05) increase in TPC and TFC from 0 to 0.4 M NaCl in stage-I. The TPC and TFC contents of the microalgae *Scenedesmus* sp. BHU1 increased by 1.5- and 1.2-fold in the 0.05 M NaCl growth culture compared to 0 M NaCl. *Scenedesmus* sp. BHU1 cells accumulated higher levels of TPC and TFC in the stage-I growth culture with 0.1, 0.15, 0.2, and 0.4 M NaCl, respectively (Table 2). Accordingly, a prior study found that *C. reinhardtii* and *A. dimorphus* significantly increased their TPC levels when exposed to salinity stress [19,39]. During stage-II cultivation, maximum TPC and TFC levels were recorded in 12-day cultures, followed by 8-day cultures (Table 2). This shows that the enhanced TPC and TFC accumulation in microalgae is mediated by salinity-induced oxidative stress. In the present study, there was a positive correlation between H_2_O_2_ and osmoprotectant molecules (proline, TPC, and TFC) in stage-I and -II cultivations (Figure S1A,B). As a result, it has become common practice to assess the intracellular levels of these parameters in order to understand the biochemical changes that occur within the cells during salinity stress. This allows for the establishment of a cause-and-effect relationship between salinity stress and cellular homeostasis during lipid synthesis in *Scenedesmus* sp. BHU1.

### 3.7. Salinity-Induced Effect on the Elemental and Mineral Composition of Scenedesmus *sp.* BHU1

**Figure 5:**
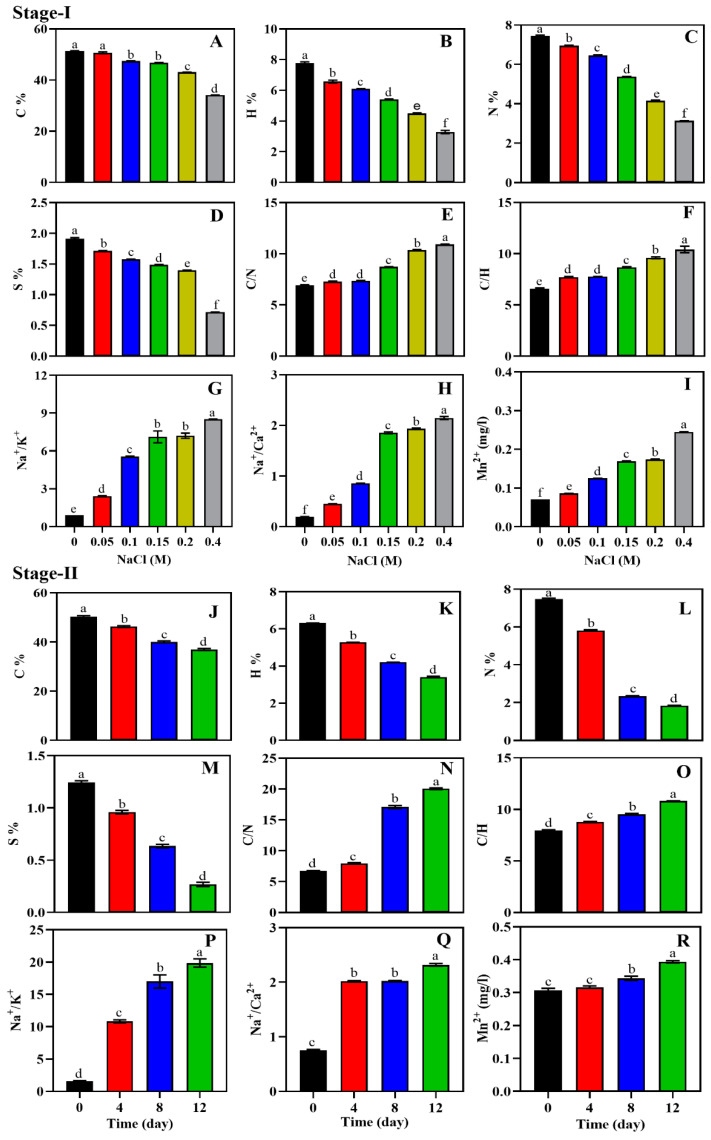
Effect of different concentrations of NaCl (0–0.4 M) during stage-I (**A**–**I**) and salinity-induced stress (0.4 M NaCl) for different durations during stage-II (**J**–**R**) on elements and mineral ions in *Scenedesmus* sp. BHU1. (**A**,**J**) Carbon; (**B**,**K**) hydrogen; (**C**,**L**) nitrogen; (**D**,**M**) sulfur; (**E**,**N**) carbon/nitrogen ratio; (**F**,**O**) carbon/hydrogen ratio; (**G**,**P**) sodium/potassium ionic ratio; (**H**,**Q**) sodium/calcium ionic ratio; (**I**,**R**) manganese ion. Bars are the mean ± standard error of mean (SEM) of three biological replicates (*n* = 3). Different lowercase alphabet letters (a–f) above the bars indicate the level of statistically significant differences, and the values followed by the same alphabet letters refer to the statistically nonsignificant differences among treatment means at the *p* < 0.05 probability threshold (Tukey’s post hoc test).

The elemental and mineral levels in the lyophilised biomass of microalgae *Scenedesmus* sp. BHU1 were influenced by the two-stage salinity stress. In two-stage cultivation, C, H, N, and S% gradually decreased significantly (*p* < 0.05) with increasing salt concentrations and time intervals (Figure 5). On the contrary, there was an increase in intracellular Na^+^ ion concentration. During stage-I cultivation, the C, H, N, and S% were higher in 0 M NaCl cultures than 0.4 M NaCl cultures (Figure 5A–D). The highest C/N and C/H ratios of the microalgal biomass were recorded in 0.4 M NaCl (Figure 5E,F). The biomass with a higher C/H ratio had a higher aromatic content with a smaller ring size, and it exhibited a higher heat release rate than the biomass with a lower C/H ratio [69]. *Scenedesmus* sp. BHU1 had a higher C/H ratio in 0.4 M NaCl-supplemented culture than other lignocellulosic and fossil fuel feedstocks, making it a potential candidate for biofuel production [70,71]. Further, *Scenedesmus* sp. BHU1 had high N (7.4%) in 0 M NaCl under stage-I cultivation (Figure 5C) that distinguished it from other lignocellulosic feedstocks, which typically contain <1% N. Reduced levels of C content with increasing salinity from 0 to 0.4 M NaCl may suggest low CO_2_ emissions during the combustion of biofuels. Additionally, during stage-I cultivation, it has been observed that the biomass treated with 0.4 M NaCl exhibits reduced levels of nitrogen and sulfur. This reduction could contribute to a decrease in the emission of harmful oxides, such as nitrogen oxides and sulfur oxides, during the conversion of biomass into biofuel. The emission of sulfur oxides is negligible in 0.4 M NaCl because of the lower sulfur content in 0.4 M NaCl (0.71 ± 0.00%) than in 0 M NaCl (1.91 ± 0.01%) under stage-I cultivation. According to [71], other green microalgal species, including *Tetradesmus obliquus* and *C. sorokiniana*, showed very similar outcomes.

The Na^+^, K^+^, Ca^2+^, and Mn^2+^ levels in the microalgal cells were considerably affected by the two-stage salt stress. Increased salinity in the growing medium had a significantly (*p* < 0.05) positive impact on the Na^+^/K^+^, Na^+^/Ca^2+^ ratio, and Mn^2+^ levels in *Scenedesmus* sp. BHU1. The Na^+^/K^+^, Na^+^/Ca^2+^ ratio, and Mn^2+^ levels in stage-I were higher in 0.4 M NaCl compared to 0 M NaCl cultures (Figure 5G–I). Accordingly, a prior study found that salt stress considerably increased the Na^+^/K^+^ and Na^+^/Ca^2+^ ratios in *Scenedesmus* sp. CCNM 1077 [21]. It has previously been reported that higher salinity reduces biomass productivity and membrane stability because of electrolyte leakage, which promotes cell death [72,73]. The maintenance of these levels is crucial for ion homeostasis and normal cell functioning. In general, ion homeostasis is maintained at high salinity by Na^+^-ATPase and H^+^-ATPase. Microalgal tolerance to salinity is primarily due to the elimination of excess Na^+^ ions through the control of their influx and efflux channels, which are triggered by the Na^+^/H^+^ antiporter [74,75]. Furthermore, [76] shows the importance of Ca^2+^ signal transduction in the increased neutral lipid accumulation by the microalgae *Chlorella* sp. C2. The research conducted in [77] revealed that lower Mn^2+^ concentrations result in lower lipid accumulation in all algal species because of Mn^2+^ redox cofactor activities. In our study, Mn^2+^ levels increased with increasing salinity stress, suggesting high lipid accumulation (Figure 5I).

The elemental and mineral levels in *Scenedesmus* sp. BHU1 during stage-II salinity stress displayed similar trends to those observed during stage-I salinity. The maximum reduction in C, H, N, and S% was observed at 12-day- compared to 0-day-grown cells (Figure 5J–M). Similar patterns of C, H, N, and S% in stage-II grown culture suggest that the elemental composition of the microalgae *Scenedesmus* sp. BHU1 is similar to stage-I. The highest C/N, C/H, Na^+^/K^+^, and Na^+^/Ca^2+^ ratios were found in cells cultivated for 12 days (Figure 5N–Q). Based on the findings, it was concluded that the microalgal *Scenedesmus* sp. BHU1 regulates cellular homeostasis by maintaining various symport and antiport channels. The current findings indicate that during both stage-I and stage-II salinity stress, there was a positive correlation between C, H, N, and S levels. Conversely, negative correlations were observed among these parameters and C/N, C/H, Na^+^/K^+^, Na^+^/Ca^2+^, and Mn^2+^ (Figure S1A,B). The addition of NaCl not only reduced the macro-elements (C, H, N, and S) of *Scenedesmus* sp. BHU1 but could also improve the quality of biofuels by lowering net-zero emissions of greenhouse gases (CO_2_ and NO_x_) and hazardous oxides (SO_x_). Our results suggest that the microalgal *Scenedesmus* sp. BHU1 could be implemented as a potential renewable and sustainable biofuel feedstock.

### 3.8. In Vivo Neutral Lipid Detection through Flow Cytometry and Fluorescent Microscopy in Scenedesmus *sp.* BHU1

**Figure 6:**
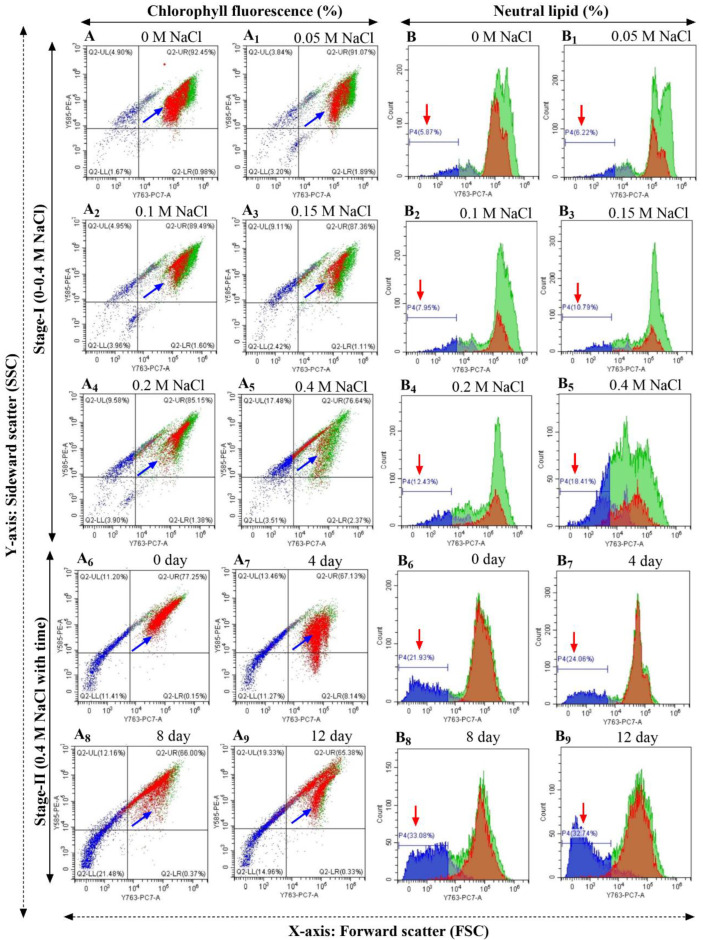
Flow cytometry plots of *Scenedesmus* sp. BHU1 cells during stage-I and -II of the two-stage cultivation. (**A**–**A_9_**) Chlorophyll fluorescence (autofluorescence signal, Y585-PE-A, 585/42, *y*-axis) and (**B**–**B_9_**) neutral lipid fluorescence (Nile red fluorescence signal, Y763-PC-7-A, 763/43, *x*-axis) were used during observation. The chlorophyll fluorescence percentage (%) is represented with blue arrows, while the neutral lipid fluorescence percentage (%) is represented with red arrows. Flow cytometry analyses were carried out using the sideward scatter (SSC, *y*-axis) and forward scatter (FSC, *x*-axis) signals of the flow cytometer CytoFLEX LX (BC47041, Beckman Coulter, Brea, CA, USA).

The effect of salinity-induced stress was evaluated using the flow cytometry (FCM) technique to further explore the variance in photosynthetic performance and neutral lipid content in *Scenedesmus* sp. BHU1. An effective phenotypic method for neutral lipid analysis is FCM-based screening using Nile red fluorescent dye. In the current work, the FCM signals in two-stage salinity stress revealed a reduction in photosynthetic activity in terms of chlorophyll fluorescence and an increase in neutral lipid content. The *y*-axis of a flow cytogram showed a sideward scatter (SSC) signal, whereas the *x*-axis showed a forward scatter (FSC) signal. In the dot plots of the FCM, *Scenedesmus* sp. BHU1 cells cultivated during stage-I showed a dose-dependent decrease in SSC and FSC signals in the quadrate-2 upper right (Q2-UR) from 92.45 to 76.64%, indicating a reduction in cell numbers (Figure 6(A–A_5_)). Based on these signals, the appearance of a new population in Q2-UR (indicated by the blue arrows) exhibit higher photosynthetic activity in 0 M NaCl culture than 0.4 M NaCl culture in stage-I. The SSC of FCM is a good indicator of cell complexity or granularity. The SSC in quadrate-2 upper left (Q2-UL) was found to be 3.5-fold higher in cells grown in 0.4 M NaCl (17.48%) than in 0 M NaCl (4.90%) culture, indicating that stress-related conditions increase the complexity of intracellular structures in stage-I cultivation (Figure 6(A,A_5_)). In contrast, population size was controlled by changes in both the FSC and SSC in Q2-UR under stage-I. During stage-II salt stress, 0-day-stressed cells had the highest photosynthetic activity (77.25%), while 4-, 8-, and 12-day-stressed cells had reduced photosynthetic activity by 67.13, 66.00, and 65.38%, respectively (Figure 6(A_6_–A_9_)). These findings showed that increased ROS accumulation, thylakoid membrane lipid peroxidation, and PSII complex degradation were the causes of the reduction in photosynthetic activity under salt stress conditions [52].

Furthermore, the cells of *Scenedesmus* sp. BHU1 were examined with FCM after being stained with Nile red dye to assess the neutral lipid accumulation under salt stress. The flow cytogram of *Scenedesmus* sp. BHU1 cells under stage-I salinity stress revealed a continuous increase in neutral lipid accumulation, indicated by the red arrows. In our investigation, 0.4 M NaCl had the maximum quantity of neutral lipid (18.41%), which was 3.1-fold higher than the culture cultivated in 0 M NaCl (5.87%) during stage-I (Figure 6(B,B_5_)). However, in stage-I cultivation, cultures exclusively subjected to high salinity stress showed an increased percentage of fluorescence intensity in stained cells. These findings were followed by an increase in cell volume, which was associated with an increase in intracellular neutral lipids. A similar result was also found in *Tetraselmis* sp. under high light intensity after 20–30 days of cultivation [78]. However, Nile red fluorescence intensity in cells cultured from 4 to 8 days gradually increased compared to 0-day cells (Figure 6(B_6_–B_8_)). These FCM results imply that cells under salt stress gradually lose chlorophyll levels while increasing neutral lipids. These findings are consistent with earlier research that focused on *Tetraselmis* sp. and *C. sorokiniana* [78,79]. Hence, in all cases, the pattern of the gravimetric analysis of the lipid content and the flow cytometry analysis of the neutral lipid results matched.

Additionally, fluorescence microscopic images confirmed the FCM findings and showed that salt stress caused a dose-dependent rise in the neutral lipid content. The degree of fluorescence was determined by the amount of neutral lipids present within the cell and the size of neutral lipid droplets. A large number of microalgal cells exhibiting red fluorescence (indicated by yellow arrows) suggested the presence of neutral lipids (Figure S2). The Nile red fluorescence intensity signal verified the existence of a significant number of neutral lipids. In a similar study, the microalgae *Chlorella* sp., *Tetraselmis* sp., and *Nannochloropsis* sp. exhibited strong associations between this fluorescence and neutral lipids [64,78,80]. Fluorescence microscopy also clearly showed a continuous rise in cell volume with improved neutral lipid granules in 0.2 and 0.4 M NaCl in stage-I (Figure S2E,F) and for 8 and 12 days in stage-II cultures (Figure S2I,J). As a result of all these salinity-related observations, *Scenedesmus* sp. BHU1 is a powerful source of neutral lipid and a reliable signifier of future renewable and sustainable biofuel feedstock.

### 3.9. Biomass Functional Group Analysis by FT-IR and Oil and Biodiesel Analysis by NMR

FT-IR spectroscopy is a spectroscopic technique that has been widely used for analysing algal biomass in a nondestructive and efficient manner, requiring only a small sample volume. Several studies have demonstrated the effectiveness of FT-IR spectroscopy in rapidly assessing lipid content without the need for lipid extraction, as well as monitoring changes in the intracellular macromolecular composition of microalgae [80,81]. To evaluate the impact of optimised cultivation conditions on lipid production in *Scenedesmus* sp. BHU1, the biomass harvested during stage-II (8 days) was selected as it exhibited the highest lipid content (38.10%).

**Figure 7:**
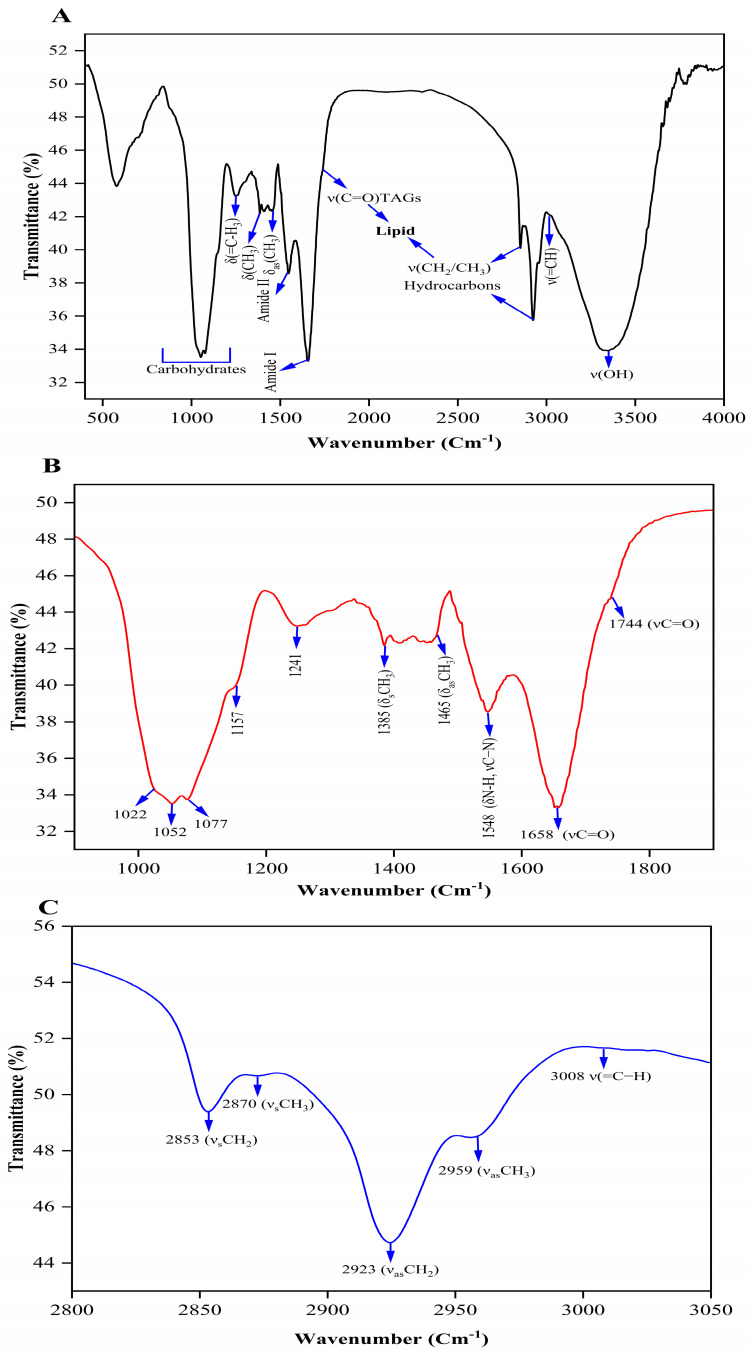
FT-IR spectra of *Scenedesmus* sp. BHU1 biomass on their 8th day of cultivation of stage-II: (**A**) spectra between 400 and 4000 cm^−1^; (**B**) spectra between 1000 and 1800 cm^−1^; (**C**) spectra between 2800 and 3050 cm^−1^.

Distinct absorption bands corresponding to various biomolecules, including carbohydrates, proteins, and lipids, were observed in the absorption spectra across the wavenumber range of 400–4000 cm^−1^ (Figure 7A). The bands detected within the 2800 to 3050 cm^−1^ region corresponded to the symmetric and asymmetric stretching vibrations of hydrocarbon groups (n(CH_2_) and n(CH_3_)) present in lipids. Additionally, a band at ~1744 cm^−1^ indicated carbonyl stretching vibrations from esters of fatty acids, specifically triacylglycerides (TAGs). The presence of amide II (~1548 cm^−1^) and amide I (~1658 cm^−1^) bands revealed characteristic vibrations of protein chains. The region between 1000 and 1250 cm^−1^ corresponded to the stretching vibrations of ν(P=O) in phospholipids and nucleic acids, as well as ν(C-O) in polysaccharides derived from carbohydrates [81]. Smaller bands at approximately 1270 cm^−1^ and 3008 cm^−1^ indicated bending vibrations δ(=C–H) and stretching vibrations ν(=C–H) of unsaturated fatty acids in algal lipids, respectively [82].

The range of 1000 to 1800 cm^−1^ displays ten distinct bands that are indicative of various biomolecules. Lipids exhibit bands at ~1385 cm^−1^ (δ_s_CH_3_), ~1465 cm^−1^ (δ_as_CH_3_), and ~1744 cm^−1^ (νC=O), while proteins show bands at ~1548 cm^−1^ (amide II δN-H, νC–N) and ~1658 cm^−1^ (amide I mainly νC=O). Carbohydrates exhibit smaller shoulder bands in the 1000–1200 cm^−1^ region, corresponding to ν(C–O–C) and ν(P=O) stretches, which are resolved as four bands at ~1022 cm^−1^, ~1052 cm^−1^, ~1077 cm^−1^, and ~1157 cm^−1^ (Figure 7B). Hydrocarbons demonstrate characteristic bands at ~2853 cm^−1^ (ν_s_CH_2_), 2870 cm^−1^ (ν_s_CH_3_), 2923 cm^−1^ (ν_as_CH_2_), and 2959 cm^−1^ (ν_as_CH_3_) (Figure 7C). Additionally, the stretching vibrations of unsaturated fatty acids, v(=CH), are clearly evident in a very small band at approximately 3008 cm^−1^ [10,82,83,84].

**Figure 8:**
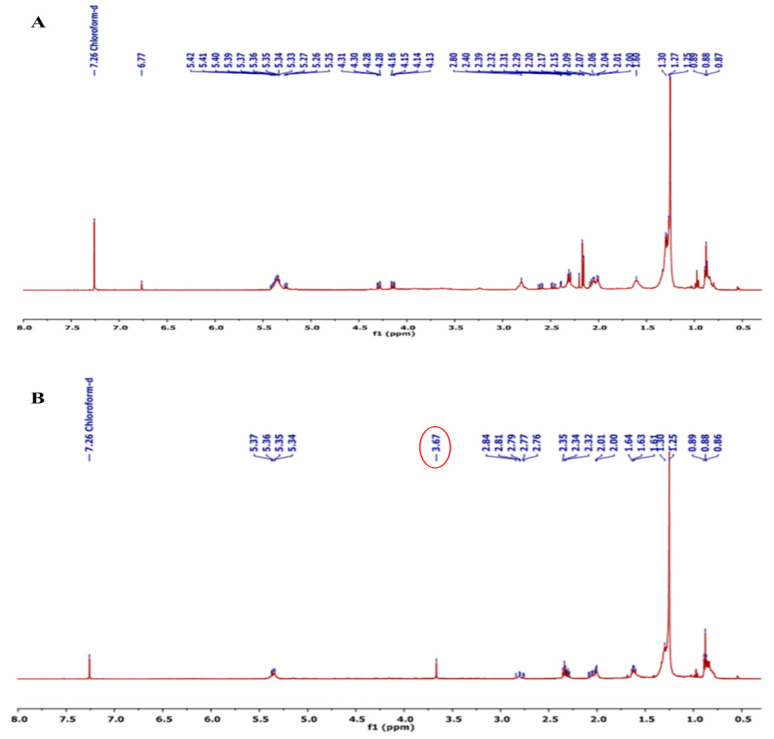
^1^H NMR spectra of (**A**) *Scenedesmus* sp. BHU1 oil and (**B**) *Scenedesmus* sp. BHU1 biodiesel at their 8th day cultivation of stage-II.

The ^1^H NMR analysis was conducted to estimate the presence of TAGs in the oil product after transesterification. In this study, an 8-day-grown culture of stage-II was utilised for NMR analysis. The ^1^H NMR spectroscopic techniques proved to be effective in assessing the transesterification process and biodiesel quality. Figure 8A,B illustrate the chemical shifts observed in the ^1^H NMR spectra of *Scenedesmus* sp. BHU1 oil and biodiesel, respectively. Notable chemical shifts include 0.88 (t, -CH_3_), 1.27 (m, -CH_3_), 1.60 (m, -CH_2_-CH_2_-), 2.29 (t, -CH_2_-COO-), and 5.33 (t, -CH=CH-). Additionally, a new strong signal peak corresponding to the methoxy group at 3.652 ppm is observed in the biodiesel spectrum (Figure 8B) but is absent in the *Scenedesmus* sp. BHU1 lipid spectrum (Figure 8A). This significant change in the biodiesel spectra of *Scenedesmus* sp. BHU1 is consistent with previous research in [10]. This finding serves as confirmation that the *Scenedesmus* sp. BHU1 accumulates a greater amount of triacylglycerides (TAGs) compared to other lipid forms. The triacylglycerides (TAGs) consist of SFAs and MUFAs, which are good components of biodiesel.

## 4. Conclusions

The current study demonstrates that *Scenedesmus* sp. BHU1 had physiochemical alterations after being exposed to NaCl. When *Scenedesmus* sp. BHU1 was grown with 0.4 M NaCl in stage-I, it produced more carbohydrate and lipid contents but less biomass. Stage-II cultures were stressed with 0.4 M NaCl for 12 days to improve the carbohydrate and lipid content. Salinity-induced stress increased the production of ROS with an increase in H_2_O_2_ levels, which led to a decreased growth rate and reduced photosynthetic activity while increasing membrane lipid peroxidation. Carotenoids, carbohydrates, lipids, proline, phenols, and flavonoids accumulated in stressed cells as osmoprotective and ROS scavenging agents. Additionally, salt stress changed the composition of elemental and mineral components, which could boost biofuel quality and result in a net-zero release of greenhouse gases. However, it also improved the biomass demands of *Scenedesmus* sp. BHU1 in terms of its hydrocarbon (C/H ratio) attributes, as well as its Na^+^/K^+^ and Na^+^/Ca^2+^ ratios, which enhanced heat release and cell homeostasis. Furthermore, flow cytometry and fluorescence microscopic analysis revealed higher neutral lipid content in cells grown with 0.4 M NaCl in stage-I and for 8 days in stage-II cultivation. The FT-IR and ^1^H NMR analyses of algal biomass and oil/biodiesel confirmed higher accumulation of triacylglycerides (TAGs) than other forms of lipids. The results of the present work provide information about the enhanced lipid content of *Scenedesmus* sp. BHU1; hence, it can be used for future biodiesel production.

## Figures and Tables

**Table 1:** Effect of different concentrations of NaCl (0–0.4 M) during stage-I and salinity-induced stress (0.4 M NaCl) for different durations during stage-II on photosynthetic pigment of *Scenedesmus* sp. BHU1.

Stage-I NaCl (M)	Chl *a* (µg/mL)	Chl *b* (µg/mL)	Caro (µg/mL)	Chl *a* + *b* (µg/mL)	Chl *a*/*b*	Caro/Chl *a* + *b*
0	6.24 ± 0.14 ^a^	4.07 ± 0.20 ^a^	0.16 ± 0.01 ^e^	10.31 ± 0.11 ^a^	1.54 ± 0.11 ^bc^	0.01 ± 0.00 ^e^
0.05	4.69 ± 0.25 ^b^	3.18 ± 0.14 ^b^	0.45 ± 0.10 ^e^	7.88 ± 0.12 ^b^	1.48 ± 0.14 ^bc^	0.05 ± 0.01 ^de^
0.1	4.17 ± 0.05 ^b^	2.74 ± 0.23 ^b^	0.85 ± 0.09 ^d^	6.91 ± 0.19 ^c^	1.54 ± 0.16 ^bc^	0.12 ± 0.01 ^d^
0.15	2.55 ± 0.22 ^c^	1.87 ± 0.15 ^c^	1.65 ± 0.10 ^c^	4.42 ± 0.23 ^d^	1.38 ± 0.19 ^c^	0.37 ± 0.03 ^c^
0.2	1.86 ± 0.06 ^cd^	1.09 ± 0.05 ^d^	2.57 ± 0.06 ^b^	2.96 ± 0.04 ^e^	1.71 ± 0.13 ^ab^	0.86 ± 0.03 ^b^
0.4	1.24 ± 0.09 ^d^	0.70 ± 0.07 ^d^	3.68 ± 0.05 ^a^	1.94 ± 0.01 ^f^	1.84 ± 0.33 ^a^	1.90 ± 0.01 ^a^
**Stage-II (0.4 M NaCl), Time (day)**						
0	6.40 ± 0.06 ^a^	3.45 ± 0.13 ^a^	0.61 ± 0.07 ^d^	9.85 ± 0.07 ^a^	1.86 ± 0.08 ^a^	0.06 ± 0.00 ^d^
4	3.61 ± 0.17 ^b^	2.30 ± 0.12 ^b^	1.56 ± 0.06 ^c^	5.92 ± 0.11 ^b^	1.58 ± 0.15 ^a^	0.26 ± 0.01 ^c^
8	2.37 ± 0.17 ^c^	2.08 ± 0.11 ^b^	2.95 ± 0.06 ^b^	4.46 ± 0.18 ^c^	1.14 ± 0.04 ^b^	0.66 ± 0.03 ^b^
12	1.28 ± 0.02 ^d^	1.42 ± 0.01 ^c^	3.63 ± 0.05 ^a^	2.70 ± 0.01 ^d^	0.90 ± 0.02 ^b^	1.34 ± 0.02 ^a^

Chl *a*: chlorophyll *a*; Chl *b*: chlorophyll *b*; Caro: carotenoids. The values are the mean ± standard error of the mean (SEM) of three biological replicates (*n* = 3). Different superscript lowercase alphabet letters (a–f) above the values indicate the levels of statistically significant differences, and the values followed by the same alphabet letters refer to the statistically nonsignificant differences among treatment means at the *p* < 0.05 probability threshold (Tukey’s post hoc test).

**Table 2:** Effect of different concentrations of NaCl (0–0.4 M) during stage-I and salinity-induced stress (0.4 M NaCl) for different durations during stage-II on stress biomarkers, osmoprotectant, and nonenzymatic antioxidant molecules of *Scenedesmus* sp. BHU1.

Stage-I NaCl (M)	H_2_O_2_ (µM mg^−1^ FW)	MDA (µM mg^−1^ FW)	Proline (µM mg^−1^ FW)	TPC (µg GAE mg^−1^ FW)	TFC (µg QE mg^−1^ FW)
0	0.06 ± 0.00 ^b^	0.04 ± 0.00 ^b^	0.06 ± 0.00 ^b^	0.13 ± 0.00 ^f^	0.05 ± 0.00 ^d^
0.05	0.10 ± 0.00 ^b^	0.04 ± 0.00 ^b^	0.08 ± 0.00 ^b^	0.20 ± 0.010 ^e^	0.06 ± 0.00 ^cd^
0.1	0.13 ± 0.01 ^b^	0.05 ± 0.00 ^b^	0.08 ± 0.00 ^b^	0.27 ± 0.00 ^d^	0.07 ± 0.00 ^bcd^
0.15	0.22 ± 0.00 ^a^	0.05 ± 0.00 ^b^	0.10 ± 0.01 ^ab^	0.32 ± 0.00 ^c^	0.08 ± 0.00 ^abc^
0.2	0.24 ± 0.00 ^a^	0.05 ± 0.00 ^b^	0.13 ± 0.00 ^a^	0.38 ± 0.00 ^b^	0.09 ± 0.00 ^ab^
0.4	0.26 ± 0.04 ^a^	0.11 ± 0.00 ^a^	0.12 ± 0.00 ^a^	0.42 ± 0.01 ^a^	0.11 ± 0.01 ^a^
**Stage-II (0.4 M NaCl), Time (day)**					
0	0.04 ± 0.01 ^b^	0.01 ± 0.00 ^b^	0.07 ± 0.01 ^b^	0.14 ± 0.00 ^c^	0.04 ± 0.00 ^c^
4	0.07 ± 0.01 ^b^	0.01 ± 0.00 ^b^	0.08 ± 0.00 ^ab^	0.24 ± 0.02 ^b^	0.06 ± 0.00 ^bc^
8	0.20 ± 0.02 ^a^	0.02 ± 0.00 ^a^	0.11 ± 0.00 ^a^	0.34 ± 0.00 ^a^	0.08 ± 0.00 ^ab^
12	0.23 ± 0.03 ^a^	0.02 ± 0.00 ^a^	0.10 ± 0.00 ^a^	0.39 ± 0.01 ^a^	0.09 ± 0.00 ^a^

H_2_O_2_: hydrogen peroxide; MDA: malonaldehyde, TPC: total phenolic content; TFC: total flavonoid content. The values are the mean ± standard error of the mean (SEM) of three biological replicates (*n* = 3). Different superscript lowercase alphabet letters (a–f) above the values indicate the level of statistically significant differences, and the values followed by the same alphabet letters refer to the statistically nonsignificant differences among treatment means at the *p* < 0.05 probability threshold (Tukey’s post hoc test).

## Data Availability

The datasets used and analysed during this study are available from the corresponding authors upon reasonable request.

## Supplementary Materials

The following supporting information can be downloaded at: https://www.mdpi.com/article/10.3390/microorganisms11082064/s1, Figure S1. Pearson correlation plot of different concentrations of NaCl (0–0.4 M) during stage-I (A) and salinity-induced stress (0.4 M NaCl) for different durations during stage-II (B) cultivation between different parameters measured to assess physiological, biochemical, stress biomarkers, and elemental responses of *Scenedesmus* sp. BHU1.A gradient in colour and size of circle could be seen between the correlation values, with large red dots exhibiting the maximum positive correlation and large blue dots exhibiting the maximum negative correlation; Figure S2. The effect of different NaCl concentrations (0–0.4 M) during stage-I (A–F) and salinity-induced stress (0.4 M NaCl) for different durations during stage-II (G–J) on neutral lipids in *Scenedesmus* sp. BHU1. The neutral lipid (NL) fluorescence is signed with yellow arrows. Fluorescent excitation emission red channel (590–650 nm) at 20X magnification was used to capture the image using fluorescent microscopy (Nikon ECLIPSE 90i, United States).
