# Supplementary material for: Salinity-Induced Physiochemical Alterations to Enhance Lipid Content in Oleaginous Microalgae Scenedesmus sp. BHU1 via Two-Stage Cultivation for Biodiesel Feedstock
Source: Microorganisms. 2023 Aug 11;11(8):2064. doi: 10.3390/microorganisms11082064 (PMC10459255; doi:10.3390/microorganisms11082064)
Supplement: Supplementary file 1 [file microorganisms-11-02064-s001.zip › microorganisms-2532677-supplementary.pdf]

*Article*

# Salinity-Induced Physiochemical Alterations to Enhance Lipid Content in Oleaginous Microalgae *Scenedesmus* sp. BHU1 Via Two-Stage Cultivation for Biodiesel Feedstock

**Rahul Prasad Singh[1], Priya Yadav[1], Ajay Kumar[2*], Abeer Hashem[3], Graciela Dolores Avila-Quezada[4], Elsayed Fathi Abd_Allah[5] and Rajan Kumar Gupta[1*]**

[1] Laboratory of Algal Research, Centre of Advanced Study in Botany, Institute of Science, Banaras Hindu University, Varanasi-221005, India
[2] Amity Institute of Biotechnology, Amity University Noida, Gautam Budha Nagar-201313, India
[3] Botany and Microbiology Department, College of Science, King Saud University, P.O. Box. 2460, Riyadh 11451, Saudi Arabia
[4] Facultad de CienciasAgrotecnológicas, Universidad Autónoma de Chihuahua, 31350, Chihuahua, Chihuahua, México
[5] Plant Production Department, College of Food and Agricultural Sciences, King Saud University, P.O. Box. 2460, Riyadh 11451, Saudi Arabia

* Correspondence: rajang.bot@bhu.ac.in, Tel. +91-9412319164; ajaykumar_bhu@yahoo.com, Tel. +91-8960639724

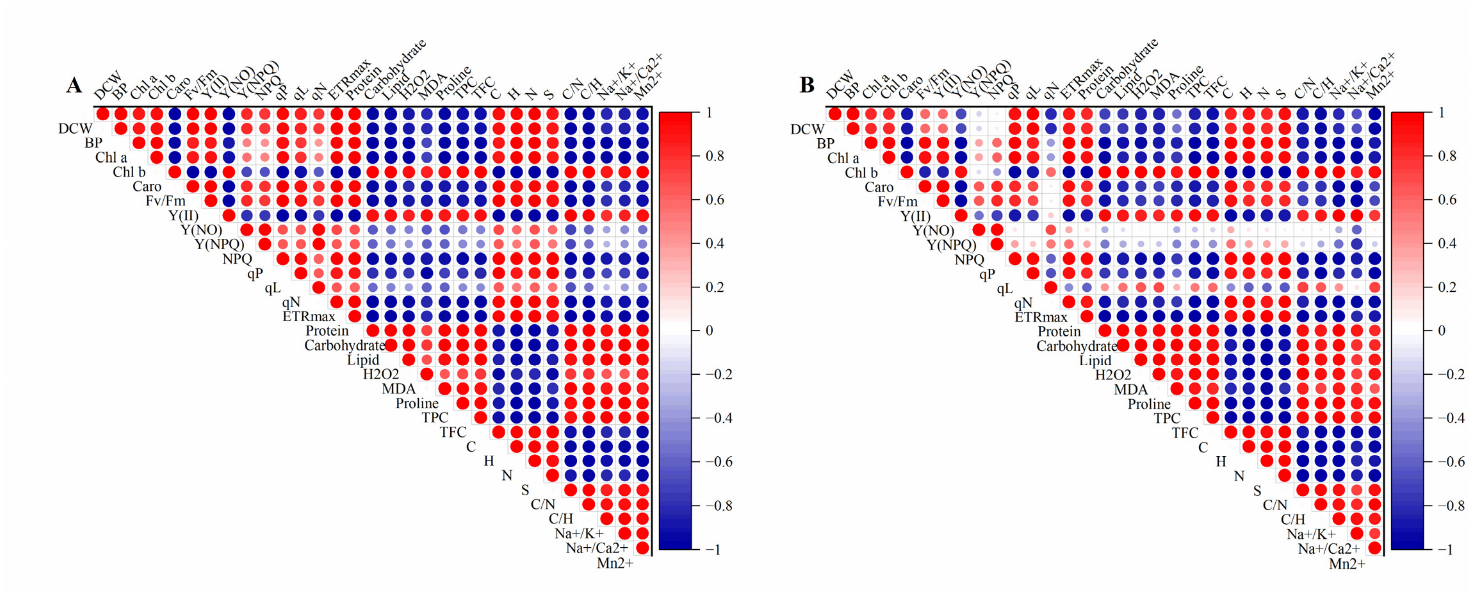


**Figure S1.** Pearson correlation plot of different concentrations of NaCl (0–0.4 M) during stage-I **(A)** and salinity-induced stress (0.4 M NaCl) for different durations during stage-II **(B)** cultivation between different parameters measured to assess physiological, biochemical, stress biomarkers, and elemental responses of *Scenedesmus* sp. BHU1.A gradient in color and size of circle could be seen between the correlation values, with large red dots exhibiting the maximum positive correlation and large blue dots exhibiting the maximum negative correlation.

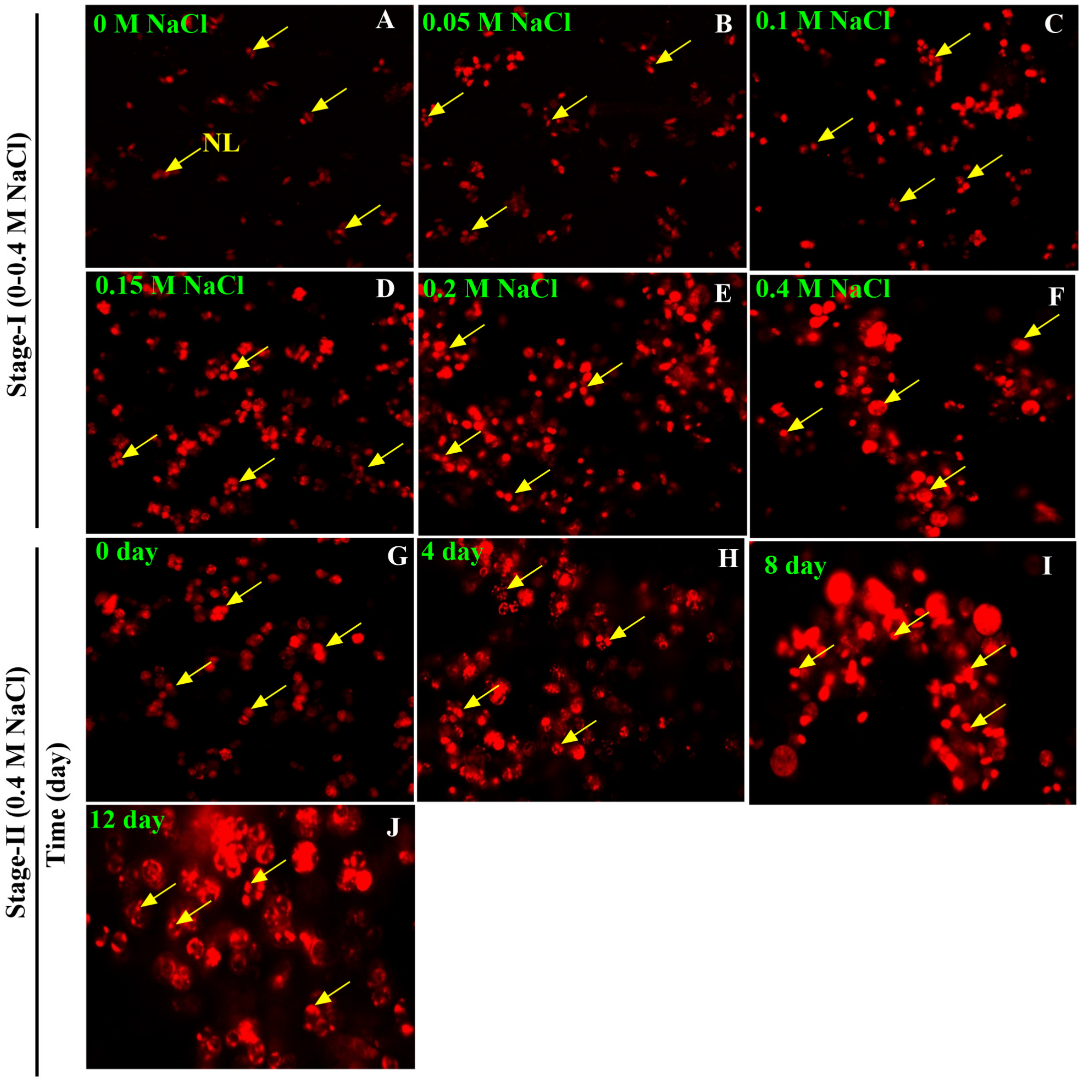


**Figure S2.** The effect of different NaCl concentrations (0–0.4 M) during stage-I **(A to F)** and salinity-induced stress (0.4 M NaCl) for different durations during stage-II **(G to J)** on neutral lipids in *Scenedesmus* sp. BHU1. The neutral lipid (NL) fluorescence is signed with yellow arrows. Fluorescent excitation emission red channel (590–650 nm) at 20X magnification was used to capture the image using fluorescent microscopy (Nikon ECLIPSE 90i, United States).
